# Reassortment Patterns in Swine Influenza Viruses

**DOI:** 10.1371/journal.pone.0007366

**Published:** 2009-10-07

**Authors:** Hossein Khiabanian, Vladimir Trifonov, Raul Rabadan

**Affiliations:** Department of Biomedical Informatics and Center for Computational Biology and Bioinformatics, Columbia University College of Physicians and Surgeons, New York, New York, United States of America; IBM Thomas J. Watson Research Center, United States of America

## Abstract

Three human influenza pandemics occurred in the twentieth century, in 1918, 1957, and 1968. Influenza pandemic strains are the results of emerging viruses from non-human reservoirs to which humans have little or no immunity. At least two of these pandemic strains, in 1957 and in 1968, were the results of reassortments between human and avian viruses. Also, many cases of swine influenza viruses have reportedly infected humans, in particular, the recent H1N1 influenza virus of swine origin, isolated in Mexico and the United States. Pigs are documented to allow productive replication of human, avian, and swine influenza viruses. Thus it has been conjectured that pigs are the “mixing vessel” that create the avian-human reassortant strains, causing the human pandemics. Hence, studying the process and patterns of viral reassortment, especially in pigs, is a key to better understanding of human influenza pandemics. In the last few years, databases containing sequences of influenza A viruses, including swine viruses, collected since 1918 from diverse geographical locations, have been developed and made publicly available. In this paper, we study an ensemble of swine influenza viruses to analyze the reassortment phenomena through several statistical techniques. The reassortment patterns in swine viruses prove to be similar to the previous results found in human viruses, both *in vitro* and *in vivo*, that the surface glycoprotein coding segments reassort most often. Moreover, we find that one of the polymerase segments (PB1), reassorted in the strains responsible for the last two human pandemics, also reassorts frequently.

## Introduction

Pandemics are epidemics that rapidly spread on a worldwide scale, caused by pathogens against which humans have no immunity that infect a large part of the population and lead to associated serious illnesses. Human influenza pandemics are caused by emerging influenza viruses from non-human reservoirs. From the three influenza pandemics of the twentieth century, the 1918 pandemic was possibly caused by an influenza virus with an avian origin [1], [2] and the other two, in 1957 and 1968, were caused by new strains that were combinations of avian and human viruses through the process of reassortment [3], [4].

There also have been many cases of swine influenza viruses infecting humans [5], [6]. In particular, in March 2009, a new human H1N1 influenza A virus of swine origin was isolated in Mexico and the United States [7], [8]. Preliminary analysis of the genome of this strain indicated that it is a descendant of common reassortant swine influenza A viruses [9]. Moreover, since 2003, a highly pathogenic H5N1 avian virus has been successfully infecting more than 400 humans with a mortality rate of 60% [10]. It is not clear whether any of these viruses will be the cause of the next human influenza pandemic, however, it is vital to understand the mechanisms behind the genomic evolution of influenza virus and its adaption to new hosts, in particular through the process of reassortment.

Influenza A virus can be found in humans and a variety of animals with aquatic birds being considered as its main reservoir. Influenza viruses do not usually transmit between different hosts. However, pigs are documented to be infected with avian and human viruses, in addition to the swine viruses. Furthermore, multiple reassortment events are found to happen under natural conditions [11]. Hence, it has been postulated that swine are the mixing vessel for inter-host influenza viruses [12].

The influenza A virus genome consists of eight single-stranded RNA segments that code for eleven known proteins. The PB2, PB1, and PA segments encode the RNA polymerase, and HA, NP, NA, and M encode hemagglutinin, nucleoprotein, neuraminidase, and the matrix proteins, respectively. Two distinct non-structural proteins are also coded by the NS segment. The subtypes of influenza A viruses are determined based on their antigenic surface glycoproteins, hemagglutinin and neuraminidase. Hemagglutinin binds to α2,3-galactose- and α2,6-galactose-linked sialic acids. The former is more preferential in avian viruses and the later in human viruses. However they are both present on the tracheal epithelium surface in pigs, making them susceptible to both avian and human viruses.

In addition to the genomic drift of influenza A virus that is caused by the high error rate in the process of replication of its genome, and the antigenic pressure on the HA and NA segments, the evolution of the virus is shaped by the reassortment process. When two different strains of influenza virus co-infect the same cell, new virions can be created that contain a mix of segments from both original strains. This phenomenon was responsible for the 1957 pandemic when the human H1N1 strain that had been circulating since 1918 reassorted to become a human H2N2 strain with new PB1, HA, and NA segments of avian origin [3], [4]. Also, in 1968, the reassortment of the PB1 and HA segments created a new human H3N2 strain which is currently co-circulating with the human H1N1 strain that reappeared in 1977 [4], [13], [14].

Swine classical H1N1 strains have been circulating in pigs since the human influenza pandemic in 1918 and were the dominant strains in the United States until 1998, when two new swine H3N2 strains were identified. These new strains were the results of a double reassortment of swine classical H1N1 with the PB1, HA, and NA segments from a human H3N2 strain, and a triple reassortment of swine classical H1N1, with the PB1, HA, and NA segments of a human H3N2 strain and the PB2 and PA segments of avian lineage [15]–[17]. So far, multiple strains of influenza virus (with various subtypes such as H1N2, H3N1, H2N3, H4N6, H5N1, etc.) have been isolated in pigs around the world, including both inter-host reassortments and whole genome adaptations of human and/or avian viruses [11], [18]–[26].

In this paper, we employ the temporally and geographically diverse information deposited in the Influenza Virus Resource of the National Center for Biotechnology Information [27] to study the reassortment phenomena in swine influenza A viruses. By integrating the information from the publicly available sequences, we investigate patterns in the reassortment events. Applying several statistical techniques, we identify the differential variability of the segments in the influenza genome and enumerate the independent reassortment events. These techniques include diversity/entropy measures of each segment and correlations between them. We find that the reassortment patterns in swine viruses are similar to the previously reported results from human viruses that HA and NA reassort more frequently than the other segments [28], [29]. Surprisingly, we also find that one of the polymerase segments, PB1, reassorts quite frequently, reiterating similar experimental results from human viruses reported by Downie [30].

## Methods

To compare the diversity within the segments of swine influenza A virus, we use strains deposited in the Influenza Virus Resource of the NCBI that have all eight segments completely sequenced. We include 150 sequences, containing 99 H1N1, 25 H1N2, 23 H3N2, and 3 H3N1 strains (see Appendix S1). For each segment, we align the sequences of their coding regions using the Smith-Waterman algorithm and calculate the normalized Hamming distances only at the third codon positions, to eliminate the effects of evolutionary pressure due to positive selection. For the M and NS segments, we only consider the coding regions of the M1 and NS1 genes, as they are the longest and the most frequently sequenced sections of the M and NS segments. Because homologous recombination is very rare or absent in influenza viruses [31], [32], this restriction does not alter the results of our analysis.

To measure the diversity of a segment *i*, we calculate *D_i_*, Rao's quadratic entropy [33], according to 
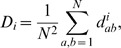
where *N* is the total number of strains in the dataset and *d^i^_ab_* is the Hamming distance between strains *a* and *b* at the third codon positions of their corresponding segment *i*. We estimate the confidence intervals for the diversity measurements via 1000 bootstrap re-samplings of the dataset.

**Figure 1:**
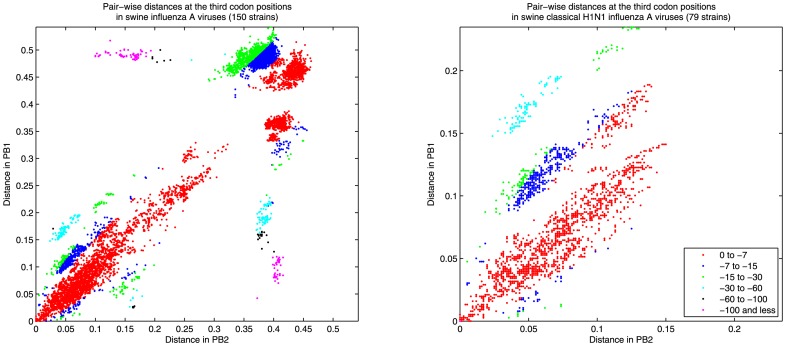
Pair-wise hamming distances at the third codon positions in PB2 vs. PB1. The colors demonstrate the logarithm of the cumulative probability for the points, among which the ones with a cumulative probability of less than 10^−7^ indicate possible reassortment events. Left: The results from 150 strains in the dataset, where there are candidates for reassortment events in both PB2 and PB1. Right: The results when the dataset is limited to the classical H1N1 strains isolated in the 70's, 80's, and 90's, where there are distinctively more candidates for reassortment events in PB1.

To find the possible reassortant strains, we primarily follow the method introduced by Rabadan *et al.* (2008), which was initially applied to complete sequences of human influenza A strains [29]. Briefly, in this method, the number of nucleotide differences between the segments of any two strains is calculated. Assuming that the segments have proportional substitution rates at the third codon positions, the differences between two segments of two strains should be proportional if the two segments have a common origin. A violation of this rule indicates that the histories of the two segments are different, i.e. there has been a reassortment event. Therefore, when the distances between two segments of different strains are plotted against each other, the points corresponding to possible reassortment events lie off the diagonal (Figure 1).

Given two strains *a* and *b* and two segments *i* and *j*, the probability to obtain hamming distances equal to *d^i^_ab_* and *d^j^_ab_* by random chance only is given by the hypergeometric distribution: 
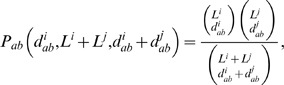
where *L_i_* and *L_j_* are the respective lengths of the segments divided by three. Hence, fixing the total distance between segments *i* and *j* of the two strains, the probability of observing a distance in segment *i* at most *d^i^_ab_* is the cumulative of the hypergeometric distribution. Maintaining the assumption of similar average substitution rates at the third codon positions in all segments, in this model the lower the cumulative probability, the more likely it is that the two segments do not have a common ancestor. To correct for multiple hypotheses testing, for every two segments of each strain we generate 100 pairs of segments by randomly permuting their third codon positions. We observe that the cumulative probabilities for distances of pairs from the generated data are at least 10^−7^. Thus, a cumulative probability of at most 10^−7^ for two given segments of two strains indicates a reassortment event.

Finally, for each of the 150 strains, we generate a list of strains with which they have low probabilities of having common ancestors, hinting to reassortment events. For further investigation of the origin of the segments, we compile a large target database of more than 10,800 strains of influenza A virus that includes all completely sequenced human and avian isolates, in addition to all swine isolates deposited in the Influenza Virus Resource of the NCBI. We use this database to compare the histories of two segments of a given swine strain. First, we align with NCBI BLAST [34] the two segments to the sequences in the target database, which precede in time the strain of interest. Second, we define the history overlap of the two segments as a function of the alignment identity in the following way. For a given alignment identity *x*, let *I_x_* be the set of target strains with which the first segment has identity at least *x*. Similarly define *J_x_* for the second segment. Then the history overlap for alignment identity *x* is the number of strains common to *I_x_* and *J_x_* over the number of strains included in either one of them. In general, low values of the history overlap function indicate distinct histories of the segments and high values correspond to common history. A decrease in the values of the history overlap function could indicate a potential fork in the lineage of one of the segments. Conversely, an increase can be the result of a merge of the lineages of the two segments, i.e. a reassortment event. Those observations allow us to confirm in an alternative and independent manner the reassortment events predicted by the hypergeometric probability analysis. The history overlap analysis is limited by the sequences present in the target database, but when enough data is available and the converging/diverging lineages are sufficiently different, it can provide a good indicator of the corresponding event.

**Figure 2:**
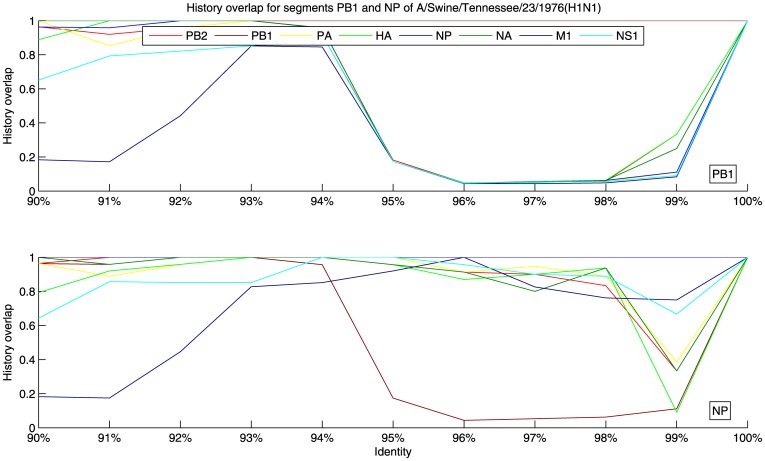
History overlap for segments PB1 and NP of the swine influenza strain A/Swine/Tennessee/23/1976. NP and all the other segments except PB1 share a common recent history, whereas the recent history of PB1 is different from the other seven segments, indicating a reassortment event at PB1. The small history overlap of M1 with PB1 and NP at lower identities can be attributed to a possible slower evolutionary rate of M1 and a fork in its lineage to a line of human viruses. The fluctuations in the history overlap of NP at 99% identity are due to small number of sample points at that level of identity.

For a demonstration of the analyses described above consider the strain A/swine/Tennesse/23/1976. When compared to A/swine/Iowa/1/1976 the hamming distance in the PB1 segments is 11% and the hamming distance in the NP segments is 3%. The cumulative hypergeometric probability of this event is less than 10^−7^, which indicates a reassortment event in at least one of those strains at either segment PB1 or NP. The history overlaps for those two segments and the rest of the segments of A/swine/Tennesse/23/1976 are shown in Figure 2. The figure shows that NP and all the other segments except PB1 share a common recent history, whereas the recent history of PB1 is different from the other seven segments. This allows us to assert that the PB1 segment of A/swine/Tennesse/23/1976 is the result of a reassortment. An interesting feature apparent in Figure 2 is that at lower identities M1 shares fewer strains with PB1 and NP. This observation can be attributed to a possible slower evolutionary rate of M1 and a fork in its lineage to a line of human viruses. Similar considerations show that the PB1 segment of A/swine/Iowa/1/1976 is also the result of a reassortment, however the PB1 segments of A/swine/Iowa/1/1976 and A/swine/Tennessee/23/1976 are from different lineages and the target database contains isolates close to the former, but not the latter.

## Results and Discussion

Viruses present an enormous diversity due to their high mutation rate, short replication time, and high number of replicates. There are several ways of measuring the diversity of a viral population: richness, evenness, Rao's entropy [33], Shannon entropy, other Renyi entropies, etc. When applied to actual viral populations, all these measures encounter similar problems: sampling bias (for instance, most of human influenza samples come from a few studies in New York State and New Zealand [27]), exponential growth and bottleneck structures of viral populations, population stratification, etc. Although the exact interpretation of these measures applied to highly structured populations is not clear, they can be used to compare the variation of diversity in different sections of the genome of a particular organism. Since similar histories imply similar diversity measures, strong differences in these measures for different sections of the genome point to their different histories.

**Figure 3:**
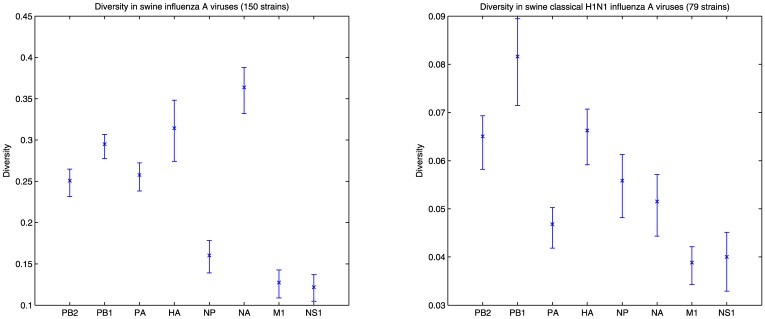
Diversity measurements in swine influenza viruses and the corresponding 95% bootstrap percentile confidence intervals. Left: Considering the 150 strains in the dataset, NA, HA, and PB1 present a higher diversity than the rest. Right: When the dataset is limited to the classical H1N1 strains isolated in the 70's, 80's, and 90's, which fixes the HA and NA variations in the population, shows a higher diversity in PB1 than the rest of the segments.

Although the third codon evolutionary rates in influenza A viruses are thought to be similar in all segments, the analysis of the genomic diversity of the strains in our dataset reveals a very inhomogeneous pattern. Figure 3 (left), shows Rao's quadratic entropy, measured at the third codon positions, for 150 swine influenza A viruses that have all 8 fully sequenced segments deposited in NCBI, along with the 95% bootstrap percentile confidence intervals. This figure indicates a statistically significant difference between NA, HA, and PB1 compared to PB2, PA, NP, M, and NS. Moreover, within a particular subtype, where the variations in HA and NA are fixed, PB1 appears as the most diverse segment. Figure 3 (right) shows the diversity in the swine classical H1N1 strains that were isolated in the 70's, 80's and 90's. This analysis shows that the eight segments do not have a common history, with PB1, HA, and NA presenting a higher level of diversity.

**Figure 4:**
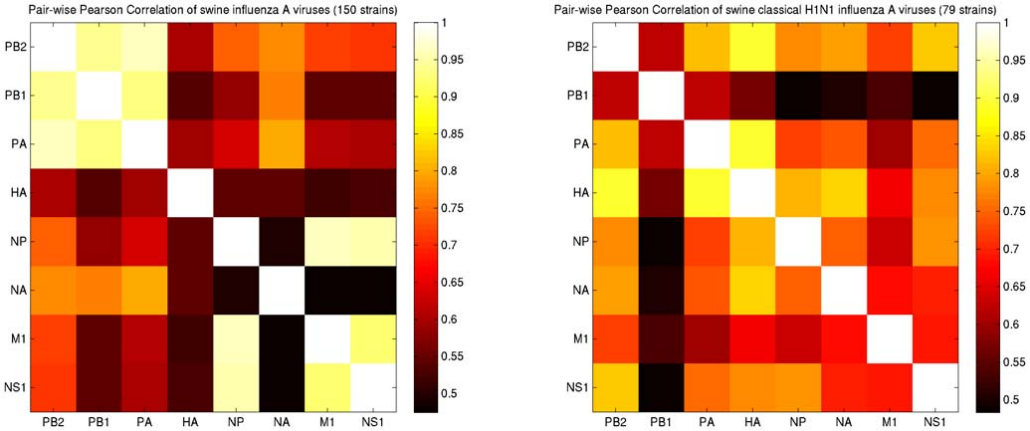
Pair-wise Pearson correlation of the distances at the third codon positions of the viral segments. Left: The HA, NA, and PB1 segments have the least correlation in regards to the rest of the segments. Right: When the HA and NA variations are fixed in the population by limiting the dataset to the classical H1N1 strains isolated in the 70's, 80's, and 90's, PB1 presents a distinctively lower correlation relative to the other segments.

We further investigate the sources of variation in diversity via the pair-wise Pearson correlation of the distances at the third codon positions of the viral segments. Correlations, linear or non-linear, or any other measure of dependence, such as mutual information, encounter the same problems as those of the measures of diversity (sampling bias, bottleneck structures, population stratification, etc.). Nonetheless, they are revealing indicators of the origins of diversity in a population. When all the 150 strains in the dataset are considered, the correlations are lower between the surface glycoprotein coding segments and the other segments. More interestingly, the PB1 segment also has a low correlation with all segments that are not polymerase coding (Figure 4, left). Especially, when the strains from a particular subtype are considered and the variations in HA and NA segments are fixed in the dataset, PB1 presents the least correlation relative to the other segments. This is evident, among the classical swine H1N1 strains isolated in the 70's, 80's and 90's (Figure 4, right).

The above observations from the diversity and pair-wise correlation measures hint to distinct evolutionary patterns in the HA, NA, and PB1 segments. To elucidate the role of the process of reassortment in these patterns, we have enumerated the independent reassortment events in swine viruses that we identify through the hypergeometric distribution analysis of Rabadan *et al.* (2008) [29] and confirm via history overlap analysis, described in the Methods section. Table 1 lists these events, represented by different strains and a simple inspection reveals the frequent role of NA, HA, and PB1 in the reassortment process. Because the reassortment events are frequent in swine viruses and the sampling is not, it is difficult to determine their exact history. However, especially in cases where there are multiple reassortments, we have attempted to identify the fully sequenced strains that are the earliest independent isolations of the reassortant viruses. As indicated in the last column of Table 1, some of the listed strains have been already published independently. In addition, for a more comprehensive list, we have included the reported reassortment events for which there are no completely sequenced isolates available, so that they could not be identified by our method. Finally, we have listed other published reassortant strains, which according to Krasnitz *et al.* (2008) either are “frozen in time” or show evidence of homologous recombination [31]. In addition to independent confirmation of the known cases of reassortment in swine viruses, our methods have succeeded in more than doubling the number of cases, as shown in Table 1.

**Table 1:** Summery of the reassortment events in swine influenza A viruses.

Year	Strain	Subtype	PB2	PB1	PA	HA	NP	NA	MP	NS	Ref.
1976	A/swine/Iowa/1/1976	H1N1	S1	S2	S1	S1	S1	S1	S1	S1	
1976	A/swine/Tennessee/15/1976	H1N1	S1	S2	S1	S1	S1	S1	S1	S1	
1976	A/swine/Tennessee/19/1976	H1N1	S1	S1	S1	S1	S1	S1	S2	S1	
1976	A/swine/Tennessee/23/1976	H1N1	S1	S2	S1	S1	S1	S1	S1	S1	
1977	A/swine/Tennessee/48/1977	H1N1	S1	S1	S2	S1	S1	S1	S2	S1	
1977	A/swine/Tennessee/61/1977	H1N1	S1	S1	S1	S1	S1	S1	S2	S1	
1977	A/swine/Tennessee/62/1977	H1N1	S1	S1	S1	S1	S1	S2	S2	S1	
1977	A/swine/Tennessee/64/1977	H1N1	S1	S2	S1	S1	S1	S1	S1	S1	
1977	A/swine/Tennessee/82/1977	H1N1	S1	S1	S1	S2	S1	S2	S1	S1	
1977	A/swine/Tennessee/96/1977	H1N1	S1	S1	S1	S1	S1	S1	S1	S2	
1979	A/swine/Minnesota/5892-7/1979	H1N1	S1	S1	S1	S1	S2	S1	S1	S1	
1981	A/swine/Ontario/6/1981	H1N1	S1	S1	S1	S1	S2	S1	S1	S1	
1986	A/swine/Iowa/1/1986	H1N1	S1	S1	S2	S2	S1	S1	S1	S1	
1988	A/swine/Wisconsin/1915/1988	H1N1	S1	S1	S1	S1	S2	S1	S1	S1	
2004	A/swine/Korea/CAN01/2004	H1N1	S1	S1	S1	S1	S1	S2	S1	S1	[25]
2004	A/swine/Spain/53207/2004	H1N1	S1	S1	S1	S2	S1	S2	S1	S3	
2007	A/swine/Ohio/24366/07	H1N1	S1	S1	S1	S2	S1	S2	S1	S1	
1998	A/swine/Italy/1521/98	H1N2	S1	S1	S1	S2	S1	S3	S1	S1	[18]
1999	A/Swine/Indiana/9K035/99	H1N2	S1	S1	S1	S2	S1	S1	S1	S1	[36]
2000	A/Swine/Minnesota/55551/00	H1N2	S1	S1	S1	S2	S1	S1	S1	S1	[37]
2004	A/swine/Zhejiang/1/2004	H1N2	S1	S1	S1	S1	S1	H	S1	S1	[19]
2005	A/swine/Cloppenburg/IDT4777/2005	H1N2	S1	S1	S1	S1	S1	S2	S1	S1	[20]
2006	A/swine/Miyazaki/1/2006	H1N2	S1	S1	S1	S1	S1	S3	S1	S1	[21]
2007	A/swine/Shanghai/1/2007	H1N2	S1	S1	S1	S2	S1	S1	S1	S1	
1998	A/Swine/Nebraska/209/98	H3N2	A	H	A	H	S	H	S	S	[36]
2001	A/swine/Spain/33601/2001	H3N2	S1	S1	S1	S2	S1	S2	S1	S1	
2003	A/swine/North Carolina/2003	H3N2	S	S	S	H1	S	H2	S	S	
2007	A/swine/Korea/CY04/2007	H3N2	S1	S1	S1	S1	S1	S1	S1	S2	[25]
2007	A/swine/Korea/CY07/2007	H3N2	S1	S1	S2	S2	S1	S1	S1	S1	[25]
	**Incompletely sequenced strains**										
1998	A/swine/North Carolina/35922/98	H3N2	S	H	S	H	S	H	S	S	[16]
2004	A/swine/MI/PU243/04	H3N1	S1	S1	S1	S1	S1	S2	S1	S1	[38]
2006	A/swine/Missouri/2124514/2006	H2N3	S1	S2	A	A	S1	A	S1	S1	[39]
	**Anomalous strains ** [**31**]										
2003	A/swine/Alberta/56626/03	H1N1	S1	S1	S2	S1	S1	S3	S1	S1	[40]
2003	A/swine/Ontario/53518/03	H1N1	S3	S3	S2	S1	S3	S1	S1	S1	[40]
2003	A/swine/Ontario/57561/03	H1N1	S1	S1	S2	S1	S3	S1	S2	S1	[40]
2004	A/swine/Ontario/48235/04	H1N2	S1	H1	S1	H2	S2	H3	S3	S3	[40]
2004	A/swine/Ontario/11112/04	H1N1	S1	H	S1	S1	S2	S1	S1	S1	[40]
2005	A/swine/Alberta/14722/2005	H3N2	S	S	S	S	S	H	S	S	[41]

Footnote: The notation is S: swine, A: avian, and H: human. The numbers distinguish different host lineages. The strains listed as anomalous are either “frozen in time” or show evidence of homologous recombination [31].

To summarize, our analyses show that not every segment of the swine influenza virus reassorts in equal fashion. In accordance with the previous results from human influenza A viruses, both *in vitro* [28] and *in vivo* [29], we find that the surface glycoproteins coding segments (HA and NA) of swine influenza viruses reassort at a higher rate as well. Perhaps, the most intriguing conclusion of our analyses is the characteristic role of one of the polymerase coding segments (PB1) that appears frequently in both inter-host and intra-host reassortment events among swine viruses and we believe that our overall analysis is the first to quantify this role. Interestingly, this is the same pattern observed in the strains responsible for the 1957 and 1968 pandemics, when human viruses also obtained PB1 segments of avian origin.

The mechanisms behind the preferential reassortments are not clear, however several hypotheses can be advanced. There is substantial evidence for biases in the packaging mechanism of the viral RNA into the virion for influenza A viruses, which can impose a selective pressure on segments that can be exchanged between strains [35]. Another constraint on the reassortment events can be associated with compensatory mutations due to interactions between the different proteins.

## Supporting Information

Appendix S1 (0.04 MB DOC)
