# Supplementary material for: Reassortment Patterns in Swine Influenza Viruses
Source: PLoS One. 2009 Oct 7;4(10):e7366. doi: 10.1371/journal.pone.0007366 (PMC2752997; doi:10.1371/journal.pone.0007366)
Supplement: Appendix S1 — (0.04 MB DOC) [file pone.0007366.s001.doc]

**Appendix S1**

The 150 sequences in this study were obtained from the Influenza Virus Resource of the National Center for Biotechnology Information [27].

Swine influenza A H1N1 strains:

1 A/swine/1931(H1N1)

2 A/swine/Alberta/56626/03(H1N1)

3 A/swine/Arizona/148/1977(H1N1)

4 A/swine/California/T9001707/1991(H1N1)

5 A/swine/Illinois/1/1975(H1N1)

6 A/swine/Iowa/1/1976(H1N1)

7 A/swine/Iowa/1/1977(H1N1)

8 A/swine/Iowa/1/1985(H1N1)

9 A/swine/Iowa/1/1986(H1N1)

10 A/swine/Iowa/1/1987(H1N1)

11 A/swine/Iowa/17672/1988(H1N1)

12 A/swine/Iowa/2/1985(H1N1)

13 A/swine/Iowa/2/1987(H1N1)

14 A/swine/Iowa/24297/1991(H1N1)

15 A/swine/Iowa/3/1985(H1N1)

16 A/swine/Iowa/31483/1988(H1N1)

17 A/swine/Iowa/4/1976(H1N1)

18 A/swine/Jamesburg/1942(H1N1)

19 A/swine/Kansas/3024/1987(H1N1)

20 A/swine/Kansas/3228/1987(H1N1)

21 A/swine/Kentucky/1/1976(H1N1)

22 A/swine/Korea/CAN01/2004(H1N1)

23 A/swine/Korea/CAS08/2005(H1N1)

24 A/swine/Kyoto/3/1979(H1N1)

25 A/swine/Maryland/23239/1991(H1N1)

26 A/swine/Memphis/1/1990(H1N1)

27 A/swine/Minnesota/24/1975(H1N1)

28 A/swine/Minnesota/27/1976(H1N1)

29 A/swine/Minnesota/5892-7/1979(H1N1)

30 A/swine/Nebraska/123/1977(H1N1)

31 A/swine/Ohio/23/1935(H1N1)

32 A/swine/Ohio/24366/07(H1N1)

33 A/swine/Ontario/11112/04(H1N1)

34 A/swine/Ontario/2/1981(H1N1)

35 A/swine/Ontario/3/1981(H1N1)

36 A/swine/Ontario/4/1981(H1N1)

37 A/swine/Ontario/53518/03(H1N1)

38 A/swine/Ontario/57561/03(H1N1)

39 A/swine/Ontario/6/1981(H1N1)

40 A/swine/Ontario/7/1981(H1N1)

41 A/swine/Saskatchewan/18789/02(H1N1)

42 A/Swine/Spain/50047/2003(H1N1)

43 A/swine/Spain/51915/2003(H1N1)

44 A/swine/Spain/53207/2004(H1N1)

45 A/swine/Tennessee/1/1975(H1N1)

46 A/swine/Tennessee/10/1976(H1N1)

47 A/swine/Tennessee/10/1977(H1N1)

48 A/swine/Tennessee/10/1978(H1N1)

49 A/swine/Tennessee/105/1977(H1N1)

50 A/swine/Tennessee/106/1977(H1N1)

51 A/swine/Tennessee/109/1977(H1N1)

52 A/swine/Tennessee/11/1978(H1N1)

53 A/swine/Tennessee/112/1977(H1N1)

54 A/swine/Tennessee/118/1977(H1N1)

55 A/swine/Tennessee/15/1976(H1N1)

56 A/swine/Tennessee/17/1976(H1N1)

57 A/swine/Tennessee/19/1976(H1N1)

58 A/swine/Tennessee/19/1977(H1N1)

59 A/swine/Tennessee/2/1978(H1N1)

60 A/swine/Tennessee/21/1977(H1N1)

61 A/swine/Tennessee/23/1976(H1N1)

62 A/swine/Tennessee/3/1976(H1N1)

63 A/swine/Tennessee/3/1978(H1N1)

64 A/swine/Tennessee/31/1977(H1N1)

65 A/swine/Tennessee/37/1977(H1N1)

66 A/swine/Tennessee/4/1978(H1N1)

67 A/swine/Tennessee/48/1977(H1N1)

68 A/swine/Tennessee/49/1977(H1N1)

69 A/swine/Tennessee/5/1978(H1N1)

70 A/swine/Tennessee/61/1977(H1N1)

71 A/swine/Tennessee/62/1977(H1N1)

72 A/swine/Tennessee/64/1977(H1N1)

73 A/swine/Tennessee/65/1977(H1N1)

74 A/swine/Tennessee/7/1976(H1N1)

75 A/swine/Tennessee/7/1978(H1N1)

76 A/swine/Tennessee/79/1977(H1N1)

77 A/swine/Tennessee/8/1978(H1N1)

78 A/swine/Tennessee/82/1977(H1N1)

79 A/swine/Tennessee/84/1977(H1N1)

80 A/swine/Tennessee/86/1977(H1N1)

81 A/swine/Tennessee/87/1977(H1N1)

82 A/swine/Tennessee/88/1977(H1N1)

83 A/swine/Tennessee/9/1978(H1N1)

84 A/swine/Tennessee/96/1977(H1N1)

85 A/swine/Virginia/670/1987(H1N1)

86 A/swine/Virginia/671/1987(H1N1)

87 A/swine/Wisconsin/1/1957(H1N1)

88 A/swine/Wisconsin/1/1961(H1N1)

89 A/swine/Wisconsin/1/1967(H1N1)

90 A/swine/Wisconsin/1/1971(H1N1)

91 A/swine/Wisconsin/11/1980(H1N1)

92 A/swine/Wisconsin/1915/1988(H1N1)

93 A/swine/Wisconsin/2/1966(H1N1)

94 A/swine/Wisconsin/2/1970(H1N1)

95 A/swine/Wisconsin/30747/1976(H1N1)

96 A/swine/Wisconsin/629/1980(H1N1)

97 A/swine/Wisconsin/641/1980(H1N1)

98 A/swine/Wisconsin/661/1980(H1N1)

99 A/swine/Wisconsin/8/1980(H1N1)

Swine influenza A H1N2 strains:

1 A/swine/Cloppenburg/IDT4777/2005(H1N2)

2 A/swine/Doetlingen/IDT4735/2005(H1N2)

3 A/swine/Guangxi/13/2006(H1N2)

4 A/Swine/Illinois/100084/01(H1N2)

5 A/Swine/Illinois/100085A/01(H1N2)

6 A/Swine/Indiana/9K035/99(H1N2)

7 A/Swine/Indiana/P12439/00(H1N2)

8 A/Swine/Iowa/930/01(H1N2)

9 A/swine/Italy/1521/98(H1N2)

10 A/swine/Korea/Asan04/2006(H1N2)

11 A/Swine/Korea/CY02/02(H1N2)

12 A/swine/Korea/Hongsong2/2004(H1N2)

13 A/swine/Korea/JL01/2005(H1N2)

14 A/swine/Korea/JL02/2005(H1N2)

15 A/swine/Korea/JL04/2005(H1N2)

16 A/swine/Korea/PZ14/2006(H1N2)

17 A/swine/Korea/PZ4/2006(H1N2)

18 A/swine/Korea/PZ7/2006(H1N2)

19 A/Swine/Minnesota/55551/00(H1N2)

20 A/swine/Miyazaki/1/2006(H1N2)

21 A/Swine/North Carolina/93523/01(H1N2)

22 A/Swine/North Carolina/98225/01(H1N2)

23 A/Swine/Ohio/891/01(H1N2)

24 A/swine/Shanghai/1/2007(H1N2)

25 A/swine/Zhejiang/1/2004(H1N2)

Swine influenza A H3N2 strains:

1 A/swine/Alberta/14722/2005(H3N2)

2 A/swine/British Columbia/28103/2005(H3N2)

3 A/Swine/Colorado/1/77(H3N2)

4 A/Swine/Iowa/533/99(H3N2)

5 A/Swine/Iowa/569/99(H3N2)

6 A/swine/Korea/CAN04/2005(H3N2)

7 A/swine/Korea/CAS07/2005(H3N2)

8 A/swine/Korea/CAS09/2006(H3N2)

9 A/swine/Korea/CY04/2007(H3N2)

10 A/swine/Korea/CY05/2007(H3N2)

11 A/swine/Korea/CY07/2007(H3N2)

12 A/swine/Korea/CY10/2007(H3N2)

13 A/swine/Korea/JNS06/2004(H3N2)

14 A/swine/Manitoba/12707/2005(H3N2)

15 A/Swine/Minnesota/593/99(H3N2)

16 A/Swine/Nebraska/209/98(H3N2)

17 A/swine/North Carolina/2003(H3N2)

18 A/swine/Ontario/33853/2005(H3N2)

19 A/swine/Spain/33601/2001(H3N2)

20 A/swine/Spain/39139/2002(H3N2)

21 A/swine/Spain/42386/2002(H3N2)

22 A/swine/Spain/54008/2004(H3N2)

23 A/Swine/Wisconsin/194/80(H3N2)

Swine influenza A H3N1 strains:

1 A/swine/IN/PU542/04(H3N1)

2 A/swine/Korea/PZ72-1/2006(H3N1)

3 A/swine/MI/PU243/04(H3N1)
